# Have geopolitics influenced decisions on American health foreign assistance efforts during the Obama presidency?

**DOI:** 10.7189/jogh.08.010417

**Published:** 2018-06

**Authors:** Vin Gupta, Alexander C Tsai, Alexandre Mason-Sharma, Eric P Goosby, Ashish K Jha, Vanessa B Kerry

**Affiliations:** 1 Harvard Global Health Institute, Harvard University, Cambridge, Massachusetts, USA; 2 Department of Pulmonary & Critical Care Medicine, Brigham & Women’s Hospital, Boston, Massachusetts, USA; 3 Department of Psychiatry, Massachusetts General Hospital, Boston, Massachusetts, USA; 4 Harvard Center for Population and Development Studies, Cambridge MA, USA; 5 Boston University, School of Medicine, Boston, Massachusetts, USA; 6 Center for Implementation Sciences, University of California, San Francisco, California, USA; 7 Harvard Global Health Institute, Cambridge, Massachusetts, USA; 8 Department of Health Policy, Harvard T.H. Chan School of Public Health, Boston, Massachusetts, USA; 9 Center for Global Health, Massachusetts General Hospital, Boston, Massachusetts, USA; 10 Department of Global Health and Social Medicine, Harvard Medical School, Boston, Massachusetts, USA

## Abstract

**Background:**

This study sought to characterize the possible relationship between US geopolitical priorities and annual decisions on health foreign assistance among recipient nations between 2009 and 2016.

**Methods:**

Data on total planned United States (US) foreign aid and health aid were collected for the 194 member nations of the World Health Organization (WHO) from publicly available databases. Trends in per-capita spending were examined between 2009 and 2016 across the six regions of the WHO (Africa, Americas, Eastern Mediterranean, Europe, Southeast Asia, and the Western Pacific). Data on US national security threats were obtained from the Council on Foreign Relations’ annual Preventive Priorities Survey. Multivariable regression models were fitted specifying planned health aid as the dependent variable and threat level of a recipient aid nation as the primary independent variable.

**Results:**

Across the aggregate 80 planned recipient countries of US health aid over the duration of the study period, cumulative planned per-capita spending was stable (US$ 0.65 in both 2009 and 2016). The number of annual planned recipients of this aid declined from 74 in 2009 to 56 in 2016 (24.3% decline), with planned allocations decreasing in the Americas, Eastern Mediterranean, and Europe; corresponding increases were observed in Africa, Southeast Asia, and the Western Pacific. Regression analyses demonstrated a dose-response, whereby higher levels of threat were associated with larger declines in planned spending (critical threat nations: b = -3.81; 95% confidence interval (CI) -5.84 to -1.78, *P* ≤ 0.001) and one-year lagged (critical threat nations: b = -3.91; 95% CI, -5.94 to -1.88, *P* ≤ 0.001) analyses.

**Conclusions:**

Higher threat levels are associated with less health aid. This is a novel finding, as prior studies have demonstrated a strong association between national security considerations and decisions on development aid.

The United States (US) is the largest contributor in absolute dollars to official development assistance annually, across both health and non-health focus areas [1]. The President’s Emergency Plan for AIDS Relief (PEPFAR) has been one of the most successful aspects of America’s development aid portfolio over the last fifteen years, even in spite of debate on its eligibility criteria and possible negative consequences on recipient nation’s health systems [2]. With greater than US$ 65 billion spent since 2003, the program has achieved considerable success, significantly reducing AIDS-associated mortality, morbidity, and mother-to-child transmission of the disease [3,4]. From a global security standpoint, PEPFAR recipient countries have often benefited from improved governance, accountability, and political stability [2]. Though documentation of direct causality remains elusive, there is growing evidence of a correlation between robust health spending and improved political systems; [5] further, these findings suggest that healthier societies are more likely to be safer and better functioning [6,7].

In the search for a financially sustainable and politically palatable policy approach to some of the world’s ongoing crises, strategic health diplomacy (SHD) may contribute a solution [2]. If PEPFAR, which receives strong bipartisan support in the American Congress, is correlated with safer and more governable societies in sub-Saharan Africa, SHD proponents (senators from both major parties, including Sen. Hatch and former Sens. Daschle and Frist) argue that US federal health aid spending could also be utilized in areas of critical geopolitical importance; for the purposes of this paper, geopolitics is defined as the study and/or management of those state-based threats relating to US national security interests.

As aid budgets are imminently forecast to become leaner [8], and given the growing desire of multilateral donors to see broad, inter-sectoral dividends from health aid [9], expanding the scope of such assistance to include more emphasis on considerations of national security may help to enhance political support and help secure future commitments to global health.

Previously, flows of official development aid (ODA) from all countries comprising the Development Assistance Committee (DAC) have been shown to be strongly associated with geopolitical considerations (the DAC consists of 30 member nations of the Organization for Economic Cooperation and Development that contribute the largest amounts of ODA (in absolute terms) annually).[10] In other words, nations of strategic interest (either because of economic or security concerns) are given priority in aid decisions relative to nations simply in need of assistance. In the case of the United States, existing data from the early-2000s show a majority of its ODA is directed to more corrupt nations (as measured by the International Country Risk Guide), whereas the nations of Scandinavia prioritize the strength of civil and governing institutions when selecting potential aid-recipients [11–15].

Associations between the magnitude of planned health aid and geopolitical considerations have not been characterized. Given the exponential growth in global health funding since the early 2000s, this study seeks to help clarify any possible relationship. The primary objective of the following analysis is to examine the relationship between a country’s geopolitical relevance to American national security interests and the magnitude of its annual anticipated US health aid package. We examine trends in planned health aid spending over the full duration of President Barack Obama’s presidency (Fiscal Years 2009-2016) to help determine whether geopolitical interests are associated with the disbursement of health aid. Planned federal health aid was examined because we believe it better reflects the prevailing political will and strategic thinking within the administration in a given year with respect to a specific country. Based on a review of recent Obama Administration policies regarding development aid for health [16], we hypothesized that there has been a weak link between national security considerations and decisions on health aid over the past 8 years.

## METHODS

### Data collection

We reviewed data from ten US independent agencies and federal executive departments that comprise America’s federally-allocated foreign aid portfolio (non-federal contributions from non-governmental organizations based in the United States were not included). Data were publicly available in online databases (http://beta.foreignassistance.gov). The agencies and departments reviewed were: Department of State (DOS), Agency for International Development (AID), Millennium Challenge Corporation (MCC), Peace Corps (PC), Inter-American Foundation (IAF), African Development Foundation (ADF), Department of the Treasury (DOT), Department of Defense (DOD), Department of Health and Human Services (HHS), and Department of Agriculture (DOA). Full data reporting for planned foreign aid during the entire study period (2009-2016) were available from the following agencies: AID, ADF, IAF, and DOT. Both PC and MCC have one year of missing data (2009 for PC, 2016 for MCC); the remaining agencies either reported partial data for all years (DOS) or had years of missing data with only partial reporting otherwise (HHS, DOA, DOD).

Between Feb 1, 2016 and April 1, 2016, data on planned spending were extracted for fiscal years 2009 through 2016. Data reporting prior to 2009 was irregular, and some gaps remained post-2009 among the ten agencies included in the study. Data on total planned US foreign aid and total planned US health aid were collected for the 194 member nations of the World Health Organization (WHO) from foreignassistance.gov. In total, 80 nations were identified for planned health aid at some point during the Obama administration. Total planned non-health foreign assistance was calculated by subtracting the total planned health aid from total planned foreign aid. Per-capita planned spending figures for US foreign aid recipient nations were calculated using population statistics as reported by the World Bank’s online database [17]. Summary statistics (median levels) on planned health spending are calculated and reported by WHO region (Africa, the Americas, Eastern Mediterranean, Europe, Southeast Asia, and the Western Pacific).

Metrics to assess the strength of governmental institutions (economic and health systems) for all nations analyzed in our study were established. These metrics were derived from descriptive data from online databases of Transparency International (TI), Institute for Health Metrics and Evaluation (IHME), and the World Bank (WB); they included metrics of corruption (from TI), [18] global burden of disease (GBD, from IHME) [19] life expectancy (as reported by the WB), and gross domestic product (GDP, from WB) [17]. Transparent governance was measured on a zero to ten scale (unitless), with higher values corresponding to more transparency (less corruption); values were available for each year in the observation period. Global burden of disease was reported in thousands of disability adjusted life-years (DALYs) per 100 000 persons due to all causes. Life expectancy was reported in years, and GDP was reported in US Dollars (USD).

To examine possible associations between planned health aid and geopolitics, we extracted data from the Council on Foreign Relations’ (CFR) annual Preventive Priorities Survey (PPS), which draws on numerous quantitative and qualitative sources to categorize nations based on their ability to affect US national security interests [20]. The sources included sending a survey to over 7000 foreign policy experts, academics, and US government officials to identify and rank the most pressing national security concerns. Nations or regions in conflict are identified on an annual basis (Appendix S1 in **Online Supplementary Document(Online Supplementary Document)**) as being the most geopolitically relevant to American foreign policy; these are then stratified into four groups based on their potential impact on US interests: critical, significant, limited, or none [20].

### Study definitions

Among US planned aid recipient nations, four different threat levels were established for the purposes of statistical analysis. The study cohorts were derived from the CFR PPS. As defined by the CFR, “critical” threat nations are those that directly threaten the US homeland, trigger US military involvement because of treaty commitments, or threaten the supply of strategic US resources. “Significant” threats are those countries of strategic importance to the US but do not involve a mutual-defense treaty commitment. “Limited” threats are defined as those that could have severe humanitarian consequences in countries of limited strategic importance to the US.

If a nation was not identified as posing a threat of any kind during the 8-year observation period, it was categorized as a “no-threat” nation. Among the nations posing some degree of threat to US interests between 2009 and 2016, their categorization was determined by the most frequent threat level posed during the study period. For example, a nation categorized as a posing a limited threat to US interests posed that specific threat level at the highest frequency over the observation period.

### Statistical analysis

We used 2-sided, paired *t* tests to compare mean statistics in 2009 and 2016 across various state economic, governance, and health financing metrics for nations earmarked to receive US health aid (n = 73 in 2009, n = 56 in 2016).

Associations between threat levels and planned US foreign health per-capita spending were subsequently analyzed using STATA software (version 14.1 SE, StataCorp, College Station, TX, USA). We fitted multivariable regression models specifying planned health aid per capita as the dependent variable, using cluster-correlated robust standard errors to account for within-country correlation over time.[21,22] As a robustness check, in a secondary analysis we lagged by one year the time-varying explanatory variables (PPS-defined threat level, time, GDP per-capita and non-health planned foreign aid per capita) to ensure that for this subset of correlations, the exposure preceded the outcome. If a country received no aid in a given year, it was included in the regression analysis with an input of “0.” In order to avoid an assumption of linear effects, GDP per capita figures were categorized into tertiles. Regression coefficients were reported in USD per-capita with corresponding 95% confidence intervals and p-values connoting levels of statistical significance (see Appendix S2 in **Online Supplementary Document(Online Supplementary Document)** for further details on regression specifications).

## RESULTS

**Figure 1:**
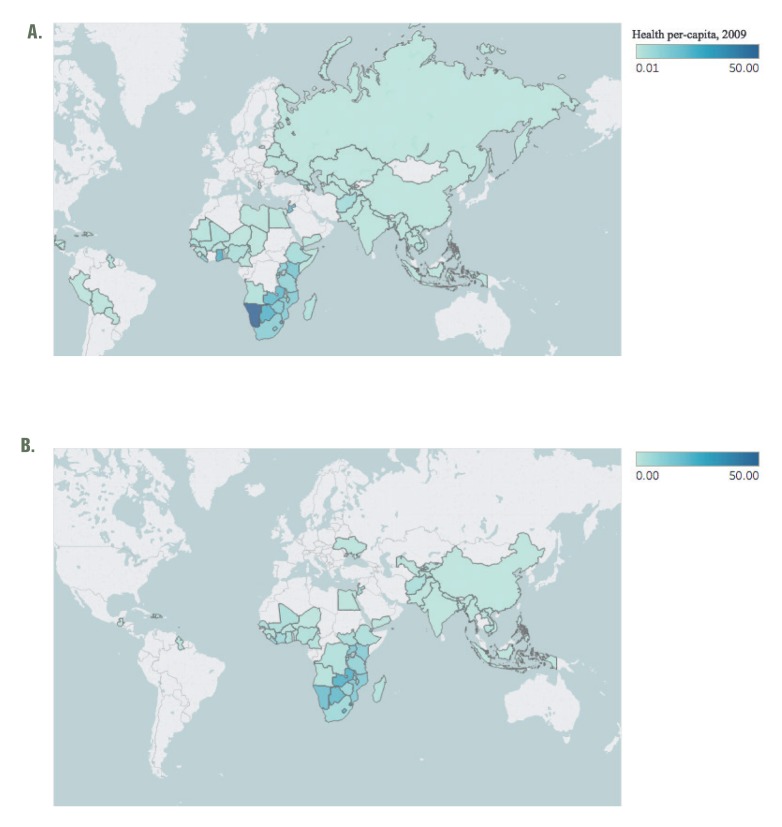
**A.** Distribution of planned US per-capita health spending globally in 2009. Data on planned US health spending in 2009 was obtained from (http://beta.foreignassistance.gov). Per-capita figures were calculated using population statistics from the World Bank. **B.** Distribution of planned US per-capita health spending globally in 2016. Data on planned US health spending in 2016 was obtained from (http://beta.foreignassistance.gov). Per-capita figures were calculated using population statistics from the World Bank.

Of the 194 nations recognized by the WHO, 80 (41.2%) were designated to receive federally directed US health aid at some point between 2009-2016. Thirty-Three African nations were a part of this cohort, representing the largest share (40.7%) of any WHO region. Thirteen (16.0%) nations from the Americas and 12 (14.8%) each from the Eastern Mediterranean and Europe were the next most represented regions, with six (7.4%) and five (6.2%) nations targeted for health aid in Southeast Asia and the Western Pacific, respectively. While 80 nations were identified for planned health spending at some point during the study period, there were fewer on an annual basis. For example, only 73 were targeted recipients in 2009, which was the highest among all years observed (**Figure 1**; panel A); this number declined to 56 by 2016 (**Figure 1**; panel B).

**Figure 2:**
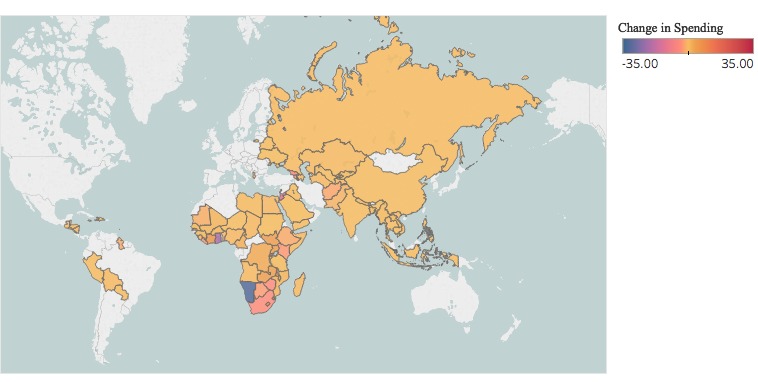
Change in per-capita planned US health spending between 2009 and 2016. Source: Foreignassistance.gov and the World Bank.

Median per-capita planned health spending across all countries remained the same in both 2009 and 2016 at US$ 0.65. Fluctuations were observed within the observation period, as the highest median planned spending levels were observed in 2011 at US$ 0.88 per-capita and the lowest in 2014 at US$ 0.58. Across regions, median per-capita spending increased among nations in the Western Pacific (US$ 0.32 in 2009, US$ 0.58 in 2016), Southeast Asia (US$ 0.13 in 2009, US$ 0.32 in 2016), and Africa (US$ 3.39 in 2009, US$ 3.72 in 2016); spending decreased in the Eastern Mediterranean (US$ 0.58 in 2009, US$ 0.11 in 2016), Europe (US$ 0.33 in 2009, US$ 0.00 in 2016), and the Americas (US$ 0.13 in 2009, US$ 0.00 in 2016). On a national level, 49 nations experienced an overall decline in planned per-capita spending levels (median decline: - US$ 0.30) between 2009 and 2016 (**Figure 2**). The largest such declines were observed in Namibia (-US$ 31.96), Ghana (-US$ 20.45), Jordan (-US$ 12.81), Rwanda (-US$ 6.50), and Georgia (-US$ 4.53). Twenty-seven nations saw an increase in planned spending (median increase: US$ 1.07) during the same period. The largest such increases were observed in Swaziland (+US$ 10.97), Lesotho (+US$ 9.38), Barbados (+US$ 7.84), Côte d'Ivoire (+US$ 6.10), and South Sudan (+US$ 4.42). All nations that exhibited an increase in per-capita spending also experienced concomitant increases in population.

**Figure 3:**
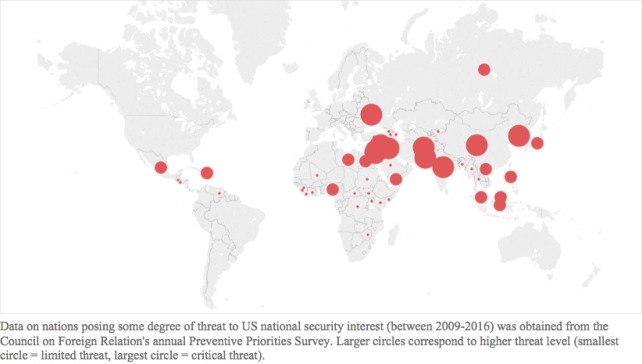
Nations posing limited, significant, or critical threats to US national security interests between 2009 – 2016. Data on nations posing some degree of threat to US national security interest (between 2009-2016) was obtained from the Council on Foreign Relations’ annual Preventive Priorities Survey. Larger circles correspond to higher threat level (smallest circle = limited threat, largest circle = critical threat).

Thirty-nine nations posed some degree of threat to US national security interests between 2009-2016 according to the PPS. The majority posed a limited threat (n = 22), while 11 nations posed significant and 6 posed critical threats (**Figure 3**).

Mean GDP (3117 vs 2561, *P* < 0.001), population (64.96 million vs 59.9 million, *P* < 0.001), life expectancy (65.5 years vs 63.5, *P* < 0.001)), and governmental transparency (3.11 vs 2.72, *P* < 0.001) were all higher among planned recipients of health aid in 2016 (n = 56) relative to 2009 (n = 73), and these findings were statistically significant (**Table 1**). Planned health (4.07 vs 3.56, *P* = 0.38) and non-health (9.70 vs 5.19, *P* = 0.02) aid per-capita and GBD (2763.5 vs 2588.1, *P* = 0.01) were both higher in 2009 vs 2016.

**Table 1:** Baseline mean characteristics (STD) of all nations targeted as recipients of US health foreign assistance in 2009 and 2016*

Variable	2009 levels	2016 levels	*P*-value
Number of planned health aid country recipients	73	56	-
Gross Domestic Product (GDP) per-capita†	2561.13 (338.74)	3117.48 (376.92)	<0.001
Global Burden of Disease‡	2763.5	2588.1	0.010
Population§	59.90 (199.10)	64.96 (209.72)	<0.001
Life expectancy‖	63.5 (0.92)	65.5 (0.84)	<0.001
Transparency Index¶	2.72 (0.11)	3.11 (0.12)	<0.001
Per-capita planned health aid**	4.07 (0.88)	3.56 (0.70)	0.380
Per-capita planned non-health aid**	9.70 (3.16)	5.19 (1.70)	0.020

STD – standard deviation

*Data on gross domestic product, life expectancy, and population figures obtained from the World Bank; Global Burden of Disease figures obtained from Institute for Health Metrics; information on Transparency obtained from Transparency International; and health aid financing data was obtained from foreignassistnace.gov.

†United States Dollars.

‡Reported in thousands of disability adjusted life-years per 100 000 persons. Last data available from 2015.

§Millions of persons.

‖Reported in years. Last data available from 2014.

¶Published by Transparency International annually. Index is on a scale of 0 to 10, with 0 connoting no transparency (high corruption) and 10 connoting perfect transparency (zero corruption).

**Data on planned health spending obtained from foreignassistance.gov.

The magnitude of planned US health aid varied across the four study cohorts over the study period. Among no-threat nations, median per-capita planned spending increased from US$ 0.84 in 2009 to an eight-year high of US$ 1.59 in 2016. Across limited-threat nations, median spending varied year-to-year though there was an overall upward trend (US$ 0.31 in 2009, US$ 0.47 in 2016, eight-year high in 2015 at US$ 0.59). Progressive declines were observed across both significant- (US$ 0.65 in 2009, US$ 0.51 in 2016) and critical-threat (US$ 0.26 in 2009, US$ 0.10 in 2016) cohorts. Over the duration of the period studied, median spending within each cohort was highest among no-threat nations (US$ 1.16 per-capita) and subsequently demonstrated a negative correlation with rising threat level (US$ 0.45 among limited-threat nations, US$ 0.42 within the significant cohort, and US$ 0.20 within the critical cohort).

In the univariate regression analysis examining the relationship between aid and geopolitics, there was a strong negative association between planned per-capita health spending and threat level across all strata: limited-threat cohort (b = -2.27; 95% CI, -4.27 to -0.26, *P* = 0.03); significant-threat cohort (b = -3.04; 95% CI -5.06 to -1.02, *P* = 0.004); and critical threat cohort (b = -2.67; 95% CI, -5.15 to -0.19, *P* = 0.04). In the one-year lagged covariate specification, similar effect sizes were observed: limited-threat cohort (b = -2.22; 95% CI, -4.24 to -0.20, *P* = 0.03); significant-threat cohort (b = -2.93; 95% CI, -5.02 to -0.84, *P* = 0.01); and critical-threat cohort (b = -2.84; 95% CI, -5.22 to -0.46, *P* = 0.02).

The multivariable regression model was adjusted for time (in years), levels of GDP per-capita, and planned US non-health per-capita spending (**Table 2**). Critical-threat countries had the largest decline in health aid spending (b = -3.81; 95% CI, -5.84 - -1.78, *P* = <0.001) over the duration of the study period, and this finding was statistically significant. The effect size increased with higher threat level, and all associations were statistically significant. In the one-year lagged analysis, similar effect sizes and trends were again observed (**Table 2**), with the largest decline in planned health spending occurring among critical-threat nations (b = -3.91; 95% CI, -5.94 - -1.88, *P* = <0.001). Non-health aid demonstrated a positive association with per-capita health spending in the primary analyses (b = 0.02; 95% CI, 0.00 to 0.05), *P* = 0.04) while escalating tertiles of GDP per-capita were associated with decreased planned health aid.

**Table 2:** Correlates of US foreign health per-capita spending

Variable	Primary analysis		Secondary analysis (lagged covariates)	
	Coefficient (95% CI)*	***P*-value**	Coefficient (95% CI)*	***P*-value**
Year	0.00 (-0.21 to 0.21)	0.99	0.02 (-0.14 to 0.21)	0.68
Threat level:†				
None	-	-	-	-
Limited	-2.26 (-4.27 to -0.26)	0.03	-2.21 (-4.21 to -0.22)	0.03
Significant	-3.33 (-5.73 to -0.93)	0.01	-3.08 (-5.46 to -0.69)	0.01
Critical	-3.81 (-5.84 to -1.78)	<0.001	-3.91 (-5.94 to -1.88)	<0.001
Non-health per-capita spending:‡				
	0.02 (0.00 to 0.05)	0.04	0.02 (0.00 to 0.04)	0.02
GDP per-capita spending:				
Lowest Tertile	–	–	–	–
Middle Tertile	-0.84 (-3.46 to 1.79)	0.53	-0.71 (-3.45 to 2.02)	0.61
Highest Tertile	0.01 (-3.78 to 3.81)	0.99	-0.32 (-3.85 to 3.20)	0.86

CI – confidence interval

^1^*Regression coefficient is reported in United States Dollars (USD) per-capita

†Threat level as determined by the Council on Foreign Relations annual Preventive Priorities Survey.

‡Reported in US$ in per-capita.

## Discussion

In this longitudinal, cross-country analysis, we observed a strong negative association between threat level and magnitude of per-capita health aid. Furthermore, a dose-response relationship was observed in the multivariable model, whereby increasing levels of threat were associated with larger effects on magnitude of planned annual health spending; these findings were statistically significant across both the primary and one-year lagged analyses.

In ancillary findings, we also demonstrate that there has been a general stability in median levels of federally-directed, planned per-capita health aid between 2009 and 2016 globally. When examining regional trends over this time period, decreased allocations to the Americas, Eastern Mediterranean, and Europe were observed while increased planned expenditures to Africa, the Western Pacific, and Southeast Asia occurred simultaneously. In total, however, the number of nations earmarked for US health foreign assistance declined by nearly 25% (n = 18) over the study period; further, a majority of nations (n = 49) saw a decline in planned per-capita health spending while 27 nations observed a corresponding increase. The median increase in per-capita spending (US$ 1.07) was nearly triple the median decline (-US$ 0.30) over the study period.

The finding that countries posing threats to American security interests (domestically or abroad) receive less in earmarked health aid annually contradicted our study hypothesis which premised no link between geopolitics and decisions on health aid allocation. The possible consequences of these trends in aid spending are likely to be observed in regions such as the Eastern Mediterranean, where the nexus of poor pre-existing health outcomes collide with strong geopolitical concerns. The retrenchment of health aid from the Eastern Mediterranean may reflect an acknowledgement on the part of US policymakers that despite high disease burden, these nations bear too much internal instability to effectively utilize health foreign assistance [23,24]. The region is home to half of the nations that pose critical threats to American national security interests (Afghanistan, Iraq, and Pakistan) and five of the eleven countries that pose significant threats (Egypt, Jordan, Lebanon, Libya, and Yemen) according to the PPS. Yet, these high-threat countries boast higher median burdens of disease and larger population sizes relative to non-threat nations. Trends in US planned aid have, however, declined over time, not increased.

Our second primary finding was that the scope of US planned health aid consolidated over the duration of the Obama administration, as a similar amount of resources were allocated to a progressively smaller group of target nations by 2016. Regions such as Europe and the Americas now have planned per-capita health spending values of zero, while the Eastern Mediterranean has seen a nearly 80% decline in median planned health aid since 2009. From a global security standpoint, the reallocation of health resources away from the aforementioned regions may in fact be destabilizing. Many nations throughout Latin America, for instance, have been most affected by Zika virus, which spread across nations with fledgling health systems and weak infrastructure for disease surveillance and associated early-warning systems [25]. The estimated global cost impact of the virus, however, was estimated at US$ 3.5 billion in 2016 [26], threatening the US directly and serving as a reminder that disregard of certain diseases may have important implications for national borders.

It is likely that poor health outcomes may precede or follow political instability within nations [27]; the argument of whether health aid can effectively address crisis and corruption indiscriminately is still being shaped. On the one hand, supporters of SHD argue that regions like the Americas or the Eastern Mediterranean benefit from increased health aid, citing the increasing evidence which correlates PEPFAR funding with the rise of more peaceful societies [5]. On the contrary, a primary obstacle to scaling up health aid is that it requires the presence of at least rudimentary systems of governance to be effective. Many of the countries that pose critical threats (Afghanistan, Syria, Iraq) lack the essential architecture of a functioning civil society making them difficult targets for planned health aid. Additionally, there are a number of factors that may influence a country’s stability including external threats (state and non-state), migrant flows, climate change, and socioeconomic instability.

Despite the aforementioned tension, data do suggest that good health can precede robust development; the rise of several East Asian nations over the past twenty years proves illustrative where health investments preceded development.[28] Improving public health systems through investments in horizontally-directed aid programs is viewed as an effective approach among developing nations [29]. Proponents of SHD argue that state stability may follow. Yet, the results of our study suggest that geopolitical considerations have not always driven decisions on health aid, representing a possible opportunity lost.

Our analysis has several strengths. In addition to filling a critical void in the literature on the landscape and possible determinants of American health aid spending, our results are based on the latest available data from the US federal government, capturing all flows of federally planned foreign aid globally. We capture trends in planned health aid across the full duration of the Obama presidency and explore novel linkages between such aid and geopolitical considerations using time-lagged, multivariable regression analyses. To our knowledge, this is the first such longitudinal and global analysis of American foreign health aid that has been conducted.

The primary limitation of this study is our inability to verify the magnitude of underreporting in planned health aid during the observation period. There are known limitations in the completeness and quality of global health financing data which have been previously described [30]. While our analysis focuses specifically on US health aid, we recognize that there are a number of data sources that can be used to track development assistance for health from non-US state actors [31]. Given the magnitude of non-state health aid, potential complementarities and/or substitution effects (whether coordinated or not) could have affected our estimates [32]. Furthermore, our analysis does not distinguish between the provision of health aid vs the management of health aid [32-34]. For example, health aid provided by the US Government but managed through global health partnerships may have a more muted or powerful effect on health diplomacy than if the aid were managed directly by the US Government.

A second potential limitation is that our analysis relies on planned health aid. There is a general consensus about the unpredictability of actual aid flows.[35-37] The 2005 Paris Declaration on Aid Effectiveness and the subsequent Accra Agenda for Action adopted at the 2008 Accra High Level Forum attempted to address this problem in part by committing donor countries to providing forward expenditure and implementation plans so that recipient countries could better incorporate aid into their medium-term plans. In this specific analysis, we focused on planned aid, which was more consistent with our conceptual goal to better understand how year-to-year strategic thinking may have influenced the obligation of health aid. However, actual aid disbursements (eg, spent aid) may be quite different and are associated with their own limitations. For example, spent aid can often reflect the implementation of projects that were actually conceived in years prior, thus introducing unpredictable lags in any assessment of how aid is deployed for strategic goals [38].

A final primary limitation is regarding the methodology that is utilized in conducting the Preventive Priorities Survey. The focus of the survey is on ranking traditional nation-state vs nation state conflicts, not potential trans-border threats such as disease outbreaks, famines, and/or economic crises. Only once in the last twenty years has a pandemic been listed as a threat (West Africa due to Ebola in 2014). This aspect of the PPS has likely resulted in an unclear amount of threat-level underestimation, reducing our statistical power to detect differences between the 4 categories of threat (eg, since the majority of nations were listed as no-threat).

In conclusion, the obligation of American health aid has remained stable in per-capita terms since 2009 though its geographic scope has narrowed over this time period. Health foreign assistance efforts have largely been consolidated within Africa while other regions that boast similarly high burdens of disease have seen their levels of earmarked health aid decline, and in some cases, approach zero. Given the emphasis of President Donald Trump to use taxpayer funds towards national and economic security goals, global health and foreign aid is less prioritized, facing severe cuts with a nearly 30% decline in recently proposed budgetary earmarks [39]. Yet, sustaining and perhaps even expanding investments in strategic health diplomacy may not only continue to save lives and burnish American soft power abroad but, in this age of rising pandemics and climate change, represent a necessary pillar of a multimodal approach to strengthening homeland security.
