# Supplementary material for: Have geopolitics influenced decisions on American health foreign assistance efforts during the Obama presidency?
Source: J Glob Health. 2018 Apr 12;8(1):010417. doi: 10.7189/jogh.08.010417 (PMC5912092; doi:10.7189/jogh.08.010417)
Supplement: Online Supplementary Document [file jogh-08-010417-s001.pdf]

Online Supplementary Document

Gupta et al. Have geopolitics influenced decisions on American health foreign assistance efforts during the Obama presidency?

J Glob Health 2018;8:010417

Appendix Exhibit S1. Countries or regions posing varying levels of threat to American national security interests, 2009-2016

Threat Level	2009	Threat Level	2010	Threat Level	2011	Threat Level	2012	Threat Level	2013	Threat Level	2014	Threat Level	2015	Threat Level	2016
Critical	Pakistan	Critical	Afghanistan	Critical	Afghanistan	Critical	North Korea	Critical	Afghanistan	Critical	Syria	Critical	Afghanistan	Critical	Israel-Palestine
Critical	Afghanistan	Critical	India-Pakistan	Critical	China	Critical	China	Critical	China	Critical	Iran	Critical	Iraq	Critical	North Korea
Critical	Iraq	Critical	Israel/Palestine	Critical	India-Pakistan	Critical	Iran	Critical	Iran	Critical	North Korea	Critical	Syria	Critical	Afghanistan
Critical	Georgia	Critical	Iraq	Critical	Mexico	Critical	Mexico	Critical	Syria	Critical	Afghanistan	Critical	Ukraine	Critical	Iraq
Critical	Israel/Palestine	Critical	Mexico	Critical	Iran	Critical	Pakistan	Critical	Pakistan	Critical	Pakistan	Critical	Israel	Critical	Libya
Critical	Iran	Critical	Pakistan	Critical	North Korea	Critical	Saudi Arabia	Significant	India-Pakistan	Critical	Yemen	Critical	North Korea	Critical	Syria
Critical	North Korea	Critical	Somalia	Critical	Pakistan	Critical	European Union	Significant	North Korea	Critical	Iraq	Critical	Russia	Critical	Egypt
Critical	China-Taiwan	Critical	Sudan	Significant	Sudan	Significant	Egypt	Significant	Mexico	Critical	Jordan	Critical	West Bank/Gaza	Critical	Turkey
Critical	Ukraine	Critical	Iran	Significant	Lebanon	Significant	India	Significant	Libya	Significant	Egypt	Significant	Nigeria	Critical	European Union
Critical	India-Pakistan	Critical	Yemen	Significant	Russia	Significant	Pakistan	Significant	Yemen	Significant	Lebanon	Significant	Significant	Significant	Mexico
				Significant	Georgia	Significant	Turkey	Significant	Bahrain	Significant	Somalia	Significant	China	Significant	Pakistan
				Significant	Saudi Arabia	Significant	Bahrain	Significant	Nigeria	Significant	Libya	Significant	Mexico	Significant	Lebanon
				Significant	Egypt	Significant	Afghanistan	Significant	Lebanon	Significant	Mexico	Significant	Pakistan	Significant	Ukraine
				Significant	Yemen	Significant	Syria	Significant	Iraq	Significant	India	Significant	Egypt	Significant	Jordan
				Significant	Haiti	Significant	Yemen	Significant	Egypt	Significant	China	Significant	Jordan	Significant	Yemen
				Significant	Iraq	Significant	Iraq	Significant	Jordan	Significant	Japan	Significant	Libya	Significant	Iran
				Significant	South Sudan	Significant	Israel	Significant	DR Congo	Significant	Nigeria	Significant	Yemen	Significant	Japan
				Limited	Somalia	Limited	Sudan	Significant	Indonesia	Significant	Central African Rep	Significant	Lebanon	Significant	China

Limited	Zimbabwe	Limited	Nigeria	Significant	Vietnam	Significant	Philippines	Significant	Turkey	Significant	Russia
Limited	DR Congo	Limited	Somalia	Significant	Philippines	Limited	Mali	Significant	Japan	Significant	Vietnam
Limited	Nigeria	Limited	Venezuela	Significant	Malaysia	Limited	Sudan	Significant	Brunei	Significant	Philippines
Limited	El Salvador	Limited	Kenya	Significant	Brunei	Limited	Turkey	Significant	Indonesia	Significant	Malaysia
Limited	Guatemala	Limited	Russia	Limited	Saudi Arabia	Limited	DR Congo	Significant	Philippines	Significant	Brunei
Limited	Burma	Limited	Georgia	Limited	Kenya	Limited	Burma	Significant	Vietnam	Limited	Nigeria
Limited	Kyrgyzstan	Limited	Libya	Limited	Turkey	Limited	Bangladesh	Significant	Malaysia	Limited	India-Pakistan
Limited	Uganda	Limited	DR Congo	Limited	Zimbabwe	Limited	Venezuela	Significant	Brunei	Limited	Democratic Republic of Congo
Limited	Ivory Coast	Limited	Kyrgyzstan	Limited	South Sudan	Limited	Armenia	Limited	Guinea	Limited	Venezuela
Limited	Thailand	Limited	Armenia	Limited	Armenia	Limited	Azerbaijan	Limited	Liberia	Limited	Central African Republic
		Limited	Azerbaijan	Limited	Azerbaijan	Limited	South Sudan	Limited	Sierra Leone	Limited	South Sudan
		Limited	South Sudan	Limited	Mali			Limited	Central African Republic	Limited	Burma
				Limited	Sudan			Limited	South Sudan	Limited	Saudi Arabia
								Limited	Thailand		
								Limited	India		
								Limited	Burma		
								Limited	Venezuela		
								Limited	Sudan		
								Limited	Armenia		
								Limited	Azerbaijan		

Appendix Exhibit S2: Main Regression Specification Estimates

The specifications of both the univariable and multivariable regressions are presented below. The one-year lagged analyses are also included for each specification. Please note that US health per-capita spending was the primary dependent variable. Threat level was the primary independent variable. The multivariable model was adjusted for time and US non-health per-capita spending.

The unit of analysis is planned per-capita US health aid. Robust standard errors are shown below in parentheses.

Univariable Regression (PRIMARY):
Number of Observations: 640
R-squared: 0.025

Variable	Coefficient (Std. Err)	T-statistic	P-value	95% CI
Threat Category				
Limited	-2.26 (1.00)	-2.25	0.027	-4.26 to -0.26
Significant	-3.02 (1.01)	-2.98	0.004	-5.04 to -1.00
Critical	-2.67 (1.25)	-2.14	0.035	-5.16 to -0.19
Constant	22.3 (163.3)	0.14	0.89	-302.6 to 347.3

Univariable Regression (ONE-YEAR LAGGED):
Number of Observations: 560
R-squared: 0.025

Variable	Coefficient (Std. Err)	T-statistic	P-value	95% CI
Threat Category				
Limited	-2.22 (1.02)	-2.18	0.032	-4.24 to -0.20
Significant	-2.93 (1.05)	-2.79	0.007	-5.02 to -0.84
Critical	-2.84 (1.25)	-2.38	0.020	-5.22 to -0.46
Constant	-54.3 (170.0)	-0.32	0.750	-392.8 to 284.1

Multivariable Regression (PRIMARY):
Number of Observations: 557
R-squared: 0.036

Variable	Coefficient (Std. Err)	T-statistic	P-value	95% CI
Threat Category				
Limited	-2.26 (1.01)	-2.25	0.027	-4.27 to -0.26
Significant	-3.33 (1.21)	-2.76	0.007	-5.73 to -0.93
Critical	-3.81 (1.02)	-3.74	<0.001	-5.84 to -1.78
Year	0.00 (0.11)	-0.01	0.99	-0.21 to 0.21
US Non-Health per-capita spending	0.02 (0.01)	2.09	0.04	0.00 to 0.05
Level of GDP per-capita	--	--	--	--
Lowest tertile	-0.84 (1.32)	-0.63	0.53	-3.46 to 1.79
Middle tertile	0.01 (1.91)	0.01	0.99	-3.78 to 3.81
Highest tertile				
Constant	6.07 (212.1)	0.03	0.98	-416.1 to 428.3

Multivariable Regression (ONE-YEAR LAGGED):
Number of Observations: 557
R-squared: 0.036

Variable	Coefficient (Std. Err)	T-statistic	P-value	95% CI
Threat Category				
Limited	-2.21 (1.00)	-2.21	0.03	-4.21 to -0.22
Significant	-3.08 (1.20)	-2.57	0.01	-5.46 to -0.69
Critical	-3.91 (1.02)	-3.83	<0.001	-5.94 to -1.88
Year	0.02 (0.10)	0.23	0.82	-0.18 to 0.23
US Non-Health per-capita spending	0.02 (0.01)	2.32	0.02	0.00 to 0.04
Level of GDP per-capita	--	--	--	--
Lowest tertile	-0.71 (1.38)	-0.52	0.61	-3.46 to 2.03
Middle tertile	-0.32 (1.77)	-0.18	0.86	-3.85 to 3.20
Highest tertile				
Constant	-44.2 (208.8)	-0.21	0.83	-460.0 to 371.5
